# Design and Prototyping of Genetically Encoded Arsenic Biosensors Based on Transcriptional Regulator AfArsR

**DOI:** 10.3390/biom11091276

**Published:** 2021-08-26

**Authors:** Salma Saeed Khan, Yi Shen, Muhammad Qaiser Fatmi, Robert E. Campbell, Habib Bokhari

**Affiliations:** 1 Department of Biosciences, Comsats University Islamabad Campus, Islamabad 45550, Pakistan; qaiser.fatmi@comsats.edu.pk; 2 Department of Chemistry, University of Alberta, Edmonton, AB T6G 2G2, Canada; yshen3@ualberta.ca (Y.S.); robert.e.campbell@ualberta.ca (R.E.C.); 3 Department of Chemistry, Graduate School of Science, The University of Tokyo, Tokyo 113-0033, Japan; 4 Department of Biosciences, Kohsar University Murree, Punjab 47200, Pakistan

**Keywords:** genetically encoded biosensor, arsenic biosensors (GEARs), FRET and FP-based arsenic biosensors

## Abstract

Genetically encoded biosensors based on engineered fluorescent proteins (FPs) are essential tools for monitoring the dynamics of specific ions and molecules in biological systems. Arsenic ion in the +3 oxidation state (As^3+^) is highly toxic to cells due to its ability to bind to protein thiol groups, leading to inhibition of protein function, disruption of protein–protein interactions, and eventually to cell death. A genetically encoded biosensor for the detection of As^3+^ could potentially facilitate the investigation of such toxicity both in vitro and in vivo. Here, we designed and developed two prototype genetically encoded arsenic biosensors (GEARs), based on a bacterial As^3+^ responsive transcriptional factor AfArsR from *Acidithiobacillus ferrooxidans*. We constructed FRET-based GEAR biosensors by insertion of AfArsR between FP acceptor/donor FRET pairs. We further designed and engineered single FP-based GEAR biosensors by insertion of AfArsR into GFP. These constructs represent prototypes for a new family of biosensors based on the ArsR transcriptional factor scaffold. Further improvements of the GEAR biosensor family could lead to variants with suitable performance for detection of As^3+^ in various biological and environmental systems.

## 1. Introduction

Arsenic and arsenic compounds ubiquitously exist in the natural environment in different forms including organic, inorganic and arsine gas. Common organic arsenic compounds include arsanilic acid (C_6_H_8_AsNO_3_), methylarsonic acid (CH_5_AsO_3_), dimethylarsinic acid (cacodylic acid, C_2_H_7_AsO_2_), and arsenobetaine (C_5_H_11_AsO_2_). The inorganic compounds, which are the most toxic, include trivalent and pentavalent compounds. Arsenic trioxide (As_2_O_3_), sodium arsenite (NaAsO_2_), and arsenic trichloride (AsCl_3_) are the most common trivalent compounds, and arsenic pentoxide (As_2_O_5_), arsenic acid (H_3_AsO_4_), and arsenates (e.g., lead arsenate PbHAsO_4_ and calcium arsenate Ca_3_(AsO_4_)_2_) are the most common pentavalent compounds [1]. Anthropogenic and naturally arsenic-contaminated groundwater and soil are the major sources of arsenic introduction into the food chain, resulting in human exposure to excessive arsenic [2]. High levels of arsenic exposure lead to health problems in humans, including many arsenicosis diseases such as skin disease, respiratory disorders, cardiovascular disorders, developmental neurotoxicity, and various cancers [3,4,5].

Methods to detect and quantify arsenic and arsenic compounds in vitro or in vivo are important to help assess arsenic contamination and its toxicity. For the in vitro detection of arsenic and its compounds, a variety of tools have been developed [6]. However, none of these detection methods provide compatibility within cell and in vivo detection of arsenic. Genetically encodable proteinaceous indicators with optical output, on the other hand, offer distinctive advantages as they can be used either in vitro or in vivo [7]. One strategy for the development of genetically encoded biosensors is to use changes in Förster resonance energy transfer (FRET) efficiency as a reporter of binding-induced conformational changes in a suitably designed fusion protein. A target molecule (analyte) binding domain (also referred to here as the sensing domain) is genetically fused between two fluorescent proteins (FPs) that serve as FRET donor (a more blue-shifted FP) and acceptor (a more red-shifted FP). Upon analyte binding to the sensing domain, a change in distance and/or orientation between the two FPs leads to alteration of FRET efficiency and a ratiometric change in fluorescence emission profile [8,9]. Another important type of genetically encoded biosensor is based on a single FP into which a sensing domain has been genetically inserted. Upon analyte binding, the sensing domain undergoes a conformational change that leads to an alteration of the FP chromophore environment, and a consequent change in the fluorescence emission intensity [10].

The use of an appropriate sensing domain in a genetically encodable biosensor can enable the biosensor to have exquisite specificity for the target molecule (that is, analyte) of interest. Appropriate sensing domains for construction of an arsenic biosensor are the arsenic binding proteins from bacterial arsenic detoxification systems. Expression of detoxification genes located in the *ars* operons is controlled by the As^3+^-responsive repressor ArsR. Upon binding to As^3+^, ArsR activates transcription of genes in the *ars* operon that encode for proteins involved in As^3+^ detoxification [11]. Among the ArsR family, three ArsR proteins have been characterized to have distinctive As^3+^ binding sites [12]. From *Escherichia coli* plasmid R773, the As^3+^ binding domain EcArsR contains three cysteines at positions Cys32, Cys34 and Cys37, which are proposed to form the As^3+^ binding site [12,13]. Recently, a FRET-based As^3+^ indicator, designated SenALiB, based on EcArsR and the ECFP/mVenus FRET pair was reported [14]. The second ArsR ortholog was discovered in *Acidithiobacillus ferrooxidans* (AfArsR). The As^3+^ binding site of AfArsR is likely composed of three cysteine residues, Cys95, Cys96, and Cys102, which are located at the flexible end of the C-terminus [15,16]. The third ArsR repressor has been identified in *Corynebacterium glutamicum* (CgArsR). In CgArsR, the three cysteines that form the binding site are located between the two dimeric subunits: Cys15 and Cys16 are located in one dimer subunit and Cys55 is located in the other [16,17]. Structural and biophysical studies of AfArsR and CgArsR have provided insight into the As^3+^ binding mode and its specificity towards As^3+^, therefore suggesting the feasibility of using such scaffolds for the design of biosensors for detecting arsenic [15,16]. In this study, we report our efforts to design, construct, and characterize prototypes of genetically encoded arsenic biosensors (GEARs) based on the *Acidithiobacillus ferrooxidans* As^3+^ responsive transcription factor, AfArsR.

## 2. Materials and Methods

### 2.1. DNA Construction and Mutagenesis

All synthetic DNA primers were purchased from Integrated DNA Technologies (IDT, Coralville, IA, USA). CloneAmp HiFi polymerase (TakaraBio, Kusatsu, Japan) was used for PCR amplification. PCR products were purified using GeneJET gel extraction kit (Thermo Scientific, Waltham, MA, USA) according to the manufacturer’s protocols. InFusion Assembly (TakaraBio, Kusatsu, Japan) was used for assembly of gene inserts and plasmid vectors. The resulting plasmids were used to transform *E. coli* DH10B electrocompetent cells (Thermo Scientific, Waltham, MA, USA). The DNA sequencing reactions were performed and analyzed at the University of Alberta Molecular Biology Service Unit by dye terminator cycle sequencing using the BigDye Terminator v3.1 Cycle Sequencing Kit (Applied Biosystems, Waltham, MA, USA). The DNA encoding AfArsR was synthesized by IDT. For construction of FRET-based GEAR-CV1, AfArsR gene was amplified using primers with overlap regions with the FRET donor and acceptor pair mCerulean3 and cpVenus, then assembled into a PCR-amplified pBAD-KIRIN1 plasmid vector. For the replacement of FRET donor and acceptor, FP mTFP1 and mCitrine gene were PCR-amplified and double digested with XhoI/KpnI and EcoR1/HindIII, respectively. The digested product was then ligated to similarly digested pBAD-GEAR-CV1 plasmid. For construction of single FP-based GEAR-G1, AfArsR gene was amplified using primers with overlap regions with GFP, then assembled into a PCR-amplified pBAD-GINKO1 plasmid vector. Site-directed mutagenesis, deletion, and linker saturation mutagenesis were performed using a QuikChange site-directed mutagenesis kit (Agilent, Santa Clara, CA, USA) following the manufacturer’s protocol. The transformed cells were spread on agar plates supplemented with 0.1 mg/mL ampicillin and 0.02% L-arabinose for 16–18 h. The colony fluorescence was inspected by using a custom-built colony screener under blue light.

### 2.2. Protein Expression and Purification

The protein was expressed as His-tagged recombinant proteins in DH10B *E. coli* cells. The culture was incubated at 37 °C for 4 h when OD achieved 0.6–0.8. Then, L-arabinose was added to a final concentration at 0.02%. Then, the culture was transferred to 30 °C for overnight incubation for a maximum of 18 h. Bacterial culture was harvested at 10,000 rpm at 4 °C for 10 min. Cell pellet was resuspended in Tris-buffered saline (TBS, 150 mM NaCl, 20 mM Tris, pH 7.5) and lysed by using sonication. The His-tagged protein from the collected supernatant was purified by affinity chromatography using Ni-NTA beads. Protein-bound beads were washed with wash buffer (20 mM imidazole, 50 mM Tris pH 7.5, 300 mM NaCl) and eluted with an elution buffer (50 mM NaH_2_PO_4_.H_2_O, 300 mM NaCl, 500 mM Imidazole). The eluted protein fractions were buffer exchanged by using a PD-10 Columns (GE Healthcare Life Sciences, Chicago, IL, USA) desalting column and then concentrated by using 10 kDa cut-off centrifuge filter columns (Amicon, Merck Millipore, Burlington, MA, USA) and stored in 1× TBS (pH 7.5). 

### 2.3. Fluorescence Measurement

For FRET-based GEARs the fluorescence spectrum was measured using a Tecan Safire2 microplate reader with excitation at 430 nm and emission from 450 nm to 600 nm. The fluorescence spectrum of the single-based GEARs was measured with excitation at 400–480 nm and emission from 480 nm to 600 nm. For measurement of *K*_d,app_ of As^3+^, purified protein was diluted into a series of buffers with As^3+^; different concentrations of sodium arsenite (Sigma-Aldrich, St. Louis, MO, USA) as the source of As^3+^ with 5 mM β-mercaptoethanol (BME) were added. The titration experiments were performed in triplicate. Data were analyzed and plotted with GraphPad Prism (8.0, GraphPad Software, San Diego, CA, USA).

### 2.4. Computational Structure Prediction of Genetically Encoded As^3+^ Biosensors

The 3D structure for GEAR-G1 was generated by using the Robetta server, which simultaneously used de novo and homology modeling methods. Structure refinement [18] and validation were carried out by using standard protocol of molecular dynamics (MD) simulation. MD simulations for the top selected model of GEAR-G1, generated by Robetta, were performed by using the GROMACS simulation package (GROMACS 2020.4) [19]. MD simulation of the protein complex GEAR-G1 was carried out for 150 ns in water using CHARMM 36 m force field for protein. The trajectory and energy files were written after every 10 ps. The system was solvated in a truncated octahedral box, containing TIP3P water molecules. The GEAR-G1 protein was centered in the simulation box within minimum distance to the box edge, with 5535 atoms overall which were neutralized by adding 7 K^+^ ions to the system. The steepest descent method was used to perform the minimization steps of 5000. To remove the steric clashes the convergence was achieved within the maximum force <1000 (KJ mol^−1^ nm^−1^). The system was equilibrated at NVT (volume and temperature at constant number of particles) and NPT (pressure and temperature at constant number of particles or molecules) ensembles for 100 ps (50,000 steps) and 1000 ps (1,000,000 steps), respectively, using time steps of 0.2 and 0.1 fs, respectively, at 300 K to ensure a fully converged system for production run. Production runs were performed at constant temperature of 300 K and at 1 atm pressure (using NPT ensemble) using weak coupling velocity-rescaling (modified Berendsen thermostat) [20] and Parrinello–Rahman algorithms [21], respectively. Relaxation times were set to τ T = 0.1 ps and τ P = 2.0 ps. All bond lengths involving hydrogen atoms were kept rigid at ideal bond lengths using the Linear Constraint Solver (lincs) algorithm, allowing for a time step of 2 fs. The Verlet scheme was used for the calculation of non-bonded interactions. Periodic boundary conditions (PBC) were used in all x, y, z directions. Interactions within a short-range cutoff of 1.2 nm were calculated in each time step. Particle mesh ewald (PME) was used to calculate the electrostatic interactions and forces to account for a homogeneous medium outside the long-range cutoff. The production was run for 150 ns for the system and for the analysis such as RMSF and PCA, only the last 100 ns was used, and the first 50 ns were discarded [22].

### 2.5. Statistical Analysis

Data are expressed as individual data points or mean ± SD. *t*-tests were used with statistical significance labeled on the figures.

## 3. Results and Discussion

### 3.1. Development of Genetically Encoded As^3+^ Biosensors Based on FRET

**Figure 1:**
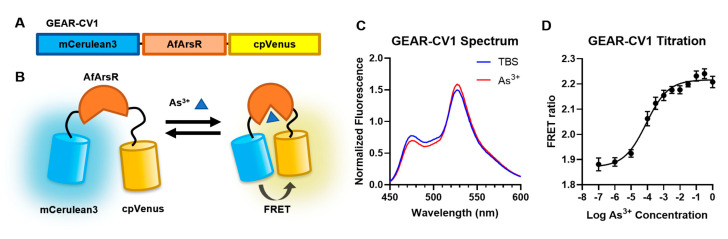
Design and characterization of the FRET-based As^3+^ biosensor prototype GEAR-CV1. (**A**) Design and gene structure of the genetically encoded As^3+^ indicator GEAR-CV1. (**B**) Schematic representation of expected FRET sensing mechanism of GEAR-CV1. (**C**) GEAR-CV1 normalized emission fluorescence spectrum (excitation at 430 nm emission scanned from 450 nm to 600 nm) in Tris-buffered saline (TBS) with (red) and without (blue) As^3+^. (**D**) FRET acceptor-to-donor fluorescence ratio (R = F_530_/F_475_) of purified GEAR-CV1 protein when titrated with As^3+^.

We first designed a FRET-based genetically encoded As^3+^ biosensor by fusing a FP FRET pair to the N- and C-termini of AfArsR (Figure 1A). We rationalized that, upon binding of As^3+^ with the three cysteine residue at position Cys95, Cys96, and Cys102, the conformational change of AfArsR would bring the fused FP FRET donor and acceptor closer together, thus increasing the FRET efficiency and resulting in a ratiometric fluorescence change (Figure 1B). We constructed the FRET-based As^3+^ biosensor by using cyan FP (CFP) mCerulean3 [23] as the FRET donor and yellow FP (YFP) variant cpVenus173 [24] as acceptor [25,26]. mCerulean3 is linked to the N-terminus of the full length AfArsR (residue 1–118) and cpVenus173 is linked to the C-terminus. The resulting construct was designated as GEAR-CV1. To characterize GEAR-CV1, we expressed and purified the protein and tested its FRET efficiency change in response to As^3+^ addition. The emission spectrum (450 nm to 600 nm) of GEAR-CV1 protein was measured before and after addition of 1 mM As^3^^+^. We observed that, in response to As^3+^, the mCerulean3 emission (~475 nm) decreased by ~10% and cpVenus (~530 nm) emission increased by ~5%, indicating an overall increase in FRET efficiency (Figure 1C). The maximum FRET acceptor and donor fluorescence emission intensity ratio (R = F_530_/F_475_) change (ΔR/R_min_) associated with the addition of As^3+^ was calculated to be 15.8 ± 0.2%. The titration results showed that there was a concentration-dependent increase in FRET ratio for GEAR-CV1 with an apparent K_d_ (K_d,app_ = concentration at half maximal change of ΔR/R_min_) of 84.9 µM (Figure 1D). These data established GEAR-CV1 as a functional FRET-based As^3+^ biosensor prototype.

**Figure 2:**
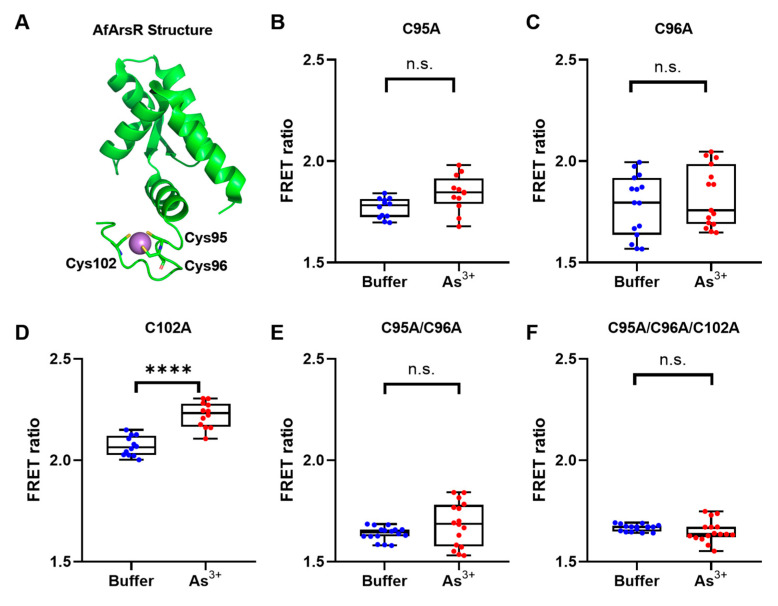
Mutational study of cysteine residues in the AfArsR arsenic binding site using GEAR-CV1. (**A**) The structure of AfArsR (PDB ID: 6J05) with cysteine residues 95, 96, and 102 (sticks) binding As^3+^ (purple sphere). FRET ratios of cysteine-mutated variants based on GEAR-CV1, in Tris-buffered saline without (blue) and with (red) 1 mM As^3+^, including single-mutants Cys95Ala (**B**) Cys96Ala (**C**), and Cys102Ala (**D**) double-mutant Cys95Ala/Cys96Ala (**E**), and triple-mutant Cys95Ala/Cys96Ala/Cys102Ala (**F**). Significant differences between pairs are indicated as **** (*p* < 0.0001), n.s. not significant (*p* > 0.05).

With the prototype biosensor constructed, we sought to use site-directed mutagenesis to investigate the potential contributions of cysteine residues in the AfArsR arsenic binding site for the sensing of As^3+^. Previous studies have demonstrated that cysteine residues 95, 96, and 102 are all involved in the As^3+^ binding (Figure 2A) [15,16]. Among these three residues, Cys95 and Cys96 have been shown to be essential for As^3+^ binding, while Cys102 is not strictly required. Specifically, the Cys102Ser mutant still maintains the ability to bind As^3+^, albeit with a reduced affinity [15]. Accordingly, we created a series of cysteine-mutated variants based on GEAR-CV1, including three single-mutants (Cys95Ala, Cys96Ala, and Cys102Ala) (Figure 2B–D), one double-mutant (Cys95Ala/Cys96Ala) (Figure 2E), and one triple-mutant (Cys95Ala/Cys96Ala/Cys102Ala) (Figure 2F). Among all these mutants, only the Cys102Ala single-mutant retained a statistically significant FRET change (7.2 ± 0.9%) upon the addition of As^3+^ (Figure 2D). All other mutants showed no response to As^3+^ (Figure 2), which is consistent with the previous biochemical study on AfArsR Cys mutations [15]. These results confirmed the distinctive roles of the cysteine residues in As^3+^ binding, and also suggested the feasibility of using a FRET-based approach for the investigation of As^3+^ binding residues. These non-binding mutants of GEAR-CV1 also demonstrate that the fluorescent proteins mCerulean3 and cpVenus do not directly bind and respond to As^3+^.

**Figure 3:**
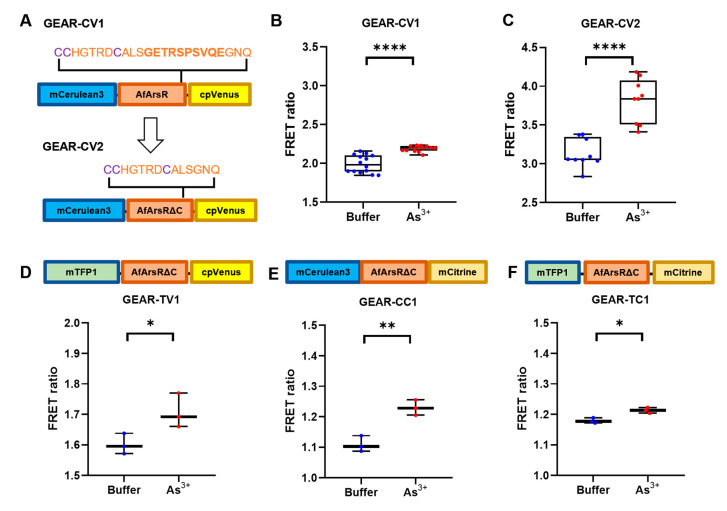
Optimization of GEAR-CV1 with structure-guided deletion and FRET donor/acceptor alteration. (**A**) Deletion of AfArsR C-terminus residue resulting in GEAR-CV2 with improved FRET ratio change. (**B**) FRET ratio of GEAR-CV1, in Tris-buffered saline without (blue) and with (red) 1 mM As^3+^. (**C**) FRET ratio of GEAR-CV2, in Tris-buffered saline without (blue) and with (red) 1 mM As^3+^. (**D**–**F**) FRET-based GEAR variants with alternative FP donor and acceptor. FRET ratios of GEAR-TV1 (mTFP1/cpVenus) (**D**), GEAR-CC1 (mCerulean3/mCitrine) (**E**), and GEAR-TC1 (mTFP1/mCitrine) (**F**), in Tris-buffered saline without (blue) and with (red) 1 mM As^3+^. Significant differences between pairs are indicated as **** (*p* < 0.0001), ** (*p* < 0.01), and * (*p* < 0.05).

We next attempted to improve the FRET ratio change (ΔR/R_min_) of GEAR-CV1. Upon inspection of the crystal structure of AfArsR, we noticed that the C-terminal region of AfArsR appears to be largely unstructured [16]. Therefore, we hypothesized that the C-terminal region might not be essential for As^3+^ binding, and that deletion of the C-terminal region in the GEAR-CV1 could effectively bring the FRET donor and acceptor closer in distance, potentially further increasing the FRET efficiency change. Accordingly, we constructed the variant GEAR-CV2 with ten C-terminal residues of AfArsR (Gly-Glu-Thr-Arg-Ser-Pro-Ser-Val-Gln-Glu) deleted (Figure 3A). Compared to the 15.8 ± 0.2% maximum ΔR/R_min_ of the template GEAR-CV1 (Figure 3B), the As^3+^ response test of GEAR-CV2 revealed the maximum ΔR/R_min_ to be 22 ± 3.5% (Figure 3C), which indicates a substantial improvement in FRET ratio change. This FRET change is also larger than the previously reported ~10% for the SenALiB As^3+^ biosensor [14]. It is worth noting that the FRET ratio of GEAR-CV2 in the As^3+^ unbound state is increased relative to GEAR-CV1 (Figure 3C), indicating that the FRET donor and acceptor are closer in proximity due to the deletion of AfArsR C-terminal residues. 

In an effort to further improve the FRET ratio change, we explored the use of alternative FP FRET donors and acceptors. We designed three additional FRET-based As^3+^ biosensor prototypes by replacing the donor and acceptor with mTFP1 and mCitrine, respectively [27,28]. Based on GEAR-CV2, we constructed GEAR-TV1 (mTFP1/cpVenus) (Figure 3D), GEAR-CC1 (mCerulean3/mCitrine) (Figure 3E), and GEAR-TC1 (mTFP1/mCitrine) (Figure 3F). In vitro characterization revealed that the FRET ratio changes ΔR/R_min_ for all these three constructs (GEAR-TV1 7 ± 2.3%, GEAR-CC1 11 ± 1.8%, GEAR-TC1 2.8 ± 0.6%) were no greater than that of GEAR-CV2 (22 ± 3.5%). Overall, the structure-guided deletion of the C-terminal residues, but not the replacements of the donor and acceptor FPs, improved the As^3+^-induced response of the FRET-based As^3+^ prototype. Accordingly, the engineered GEAR-CV2 is a promising template for further genetically encoded FRET biosensor development leading to eventual application for As^3+^ detection.

### 3.2. Development of Genetically Encoded As^3+^ Biosensors Based on a Single FP

**Figure 4:**
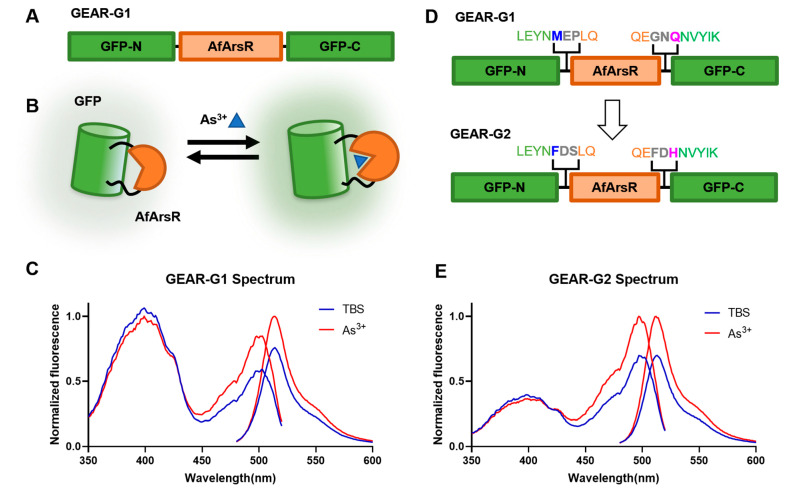
Design and engineering of single FP-based As^3+^ biosensor prototypes GEAR-G1 and GEAR-G2. (**A**) Design and DNA construction of the genetically encoded As^3+^ indicator GEAR-G1. (**B**) Schematic representation of the sensing mechanism of GEAR-G1. (**C**) GEAR-G1 normalized excitation and emission fluorescence spectrum in Tris-buffered saline (TBS) with (red) and without (blue) As^3+^. (**D**) Rational optimization of residues 145 and 148 of GFP (blue and magenta residues), the linker regions (grey residues) for improved single FP-based As^3+^ biosensor prototype GEAR-G2. (**E**) GEAR-G2 normalized excitation and emission fluorescence spectrum in Tris-buffered saline (TBS) with (red) and without (blue) As^3+^.

Following on from our success at constructing the prototype FRET-based GEAR-CV1 and GEAR-CV2, we further explored the possibility of constructing a single FP-based As^3+^ biosensor using AfArsR as a binding domain. We designed and constructed a genetically encoded As^3+^ biosensor based on a single green FP (GFP). We used an insertion-type biosensor topology, in which the intact AfArsR domain was inserted into GFP in close proximity with the chromophore (Figure 4A). Specifically, residues 146–147 of GFP were replaced with AfArsR, with two residue-long linkers at both connection points. The conformational change of AfArsR upon binding to As^3+^ was expected to alter the chromophore environment, thus changing the fluorescence emission intensity (Figure 4B). This topology has been previously reported for the construction of Ca^2+^, glutamate, K^+^, and citrate biosensors [26,29,30,31] among many others. We found that insertion of intact AfArsR into the GFP scaffold resulted in a prototype green fluorescent indicator, designated as GEAR-G1. Upon addition of 1 mM As^3+^ to purified GEAR-G1, the fluorescence emission intensity change (Δ*F*/*F*_0_) is 31.6% (Figure 4C). 

To improve the response of GEAR-G1 biosensor toward As^3+^, we focused our rational engineering efforts on the linkers that connect GFP to the AfArsR domain, and the residues of GFP (residue 145 as numbered in GFP = residue 146 as numbered in GEAR-G1; and residue 148 as numbered in GFP = residue 263 as numbered in GEAR-G1) that are connected to the linkers (Figure 4D). Based on structural and mechanistic insights gained from analyzing highly engineered single FP-based biosensors [32], the sequence consensus suggested that mutating the residues at Met146 and Gln263 to Phe146 and His263, respectively, could potentially improve the biosensor. We thus performed site-directed mutagenesis to obtain GEAR-G1-Met146Phe/Gln263His. Starting from this variant, we optimized the linker regions by randomizing linker1 (Glu147-Pro148) and linker2 (Gly261-Asn262) using saturation mutagenesis. Upon screening the linker mutagenesis libraries, we identified GEAR-G1-Met146Phe/Glu147Asp/Pro148Ser/Gly261Phe/Asn262Asp/Gln263His with optimized linker sequences. This linker optimized variant, designated as GEAR-G2, has a maximum Δ*F*/*F*_0_ of 44.9% upon binding of As^3+^ (Figure 4E). Altogether, the rational approaches of consensus engineering and linker optimization substantially improved the GEAR-G1 prototype biosensor in terms of As^3+^-induced fluorescent emission intensity change. The development and further improvement of the prototype GEAR-G2 As^3+^ biosensor provides strong support for the conclusion that the ArsR scaffold can be used for the construction of genetically encoded single FP-based indicators. Nonetheless, the established GEAR-G2 biosensor exhibits relatively low sensitivity, as expected for a prototype biosensor. Further optimization efforts are likely to eventually lead to high-performance biosensors with greater sensitivity and improved utility.

### 3.3. Computational Structure Prediction of Genetically Encoded As^3+^ Biosensors

**Figure 5:**
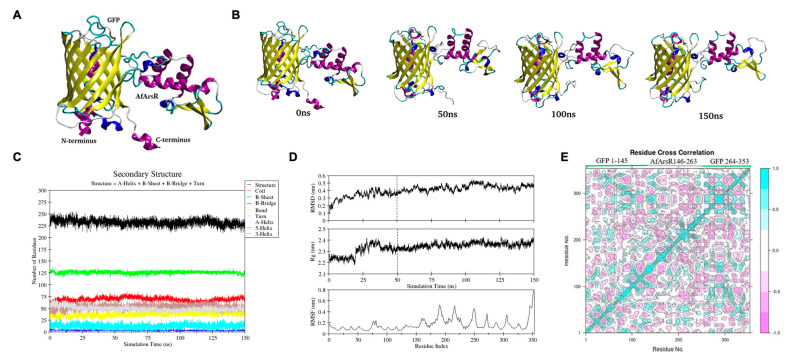
(**A**) Structure of GEAR G1 predicted by Robetta. (**B**) Snapshots of structure of GEAR-G1 at different time scales of MD simulation. Yellow, purple, blue, and cyan/white colors depict various secondary structure elements such as β-sheets, α-helix, short α-helices, and coil/turns, respectively. (**C**) Dictionary of Secondary Structure of GEAR-G1 protein (DSSP), as obtained from 150 ns of MD simulation. (**D**) RMSD, Rg, and RMSF plots as calculated from the α-carbons of the GEAR-G1 protein. The RMSF plot has been generated for the last 100 ns of the trajectory. Residues 146–263 correspond to AfArsR, and residues 1–145 and 264–353 correspond to GFP. (**E**) Dynamic cross correlation matrix map of the trajectory for GEAR-G1 protein complex. The value ranges from +1 (cyan color) to −1 (purple color). Positive values represent positive inter-residual correlation, while negative values represent negative inter-residual correlation. The map depicts that both a positive and negative inter-residual correlation exists in the atoms of GEAR-G1 protein, which is essential for its function.

To obtain further insight into the structure and mechanism of the GEAR-G1 biosensor, we employed computational structure prediction and simulations. We used multiple online servers including i-TASSER, SWISS MODEL, Phyre2, and Rosetta [33,34,35,36] to generate models of the protein where they were compared and selected. Our selection criteria for the model was based on the perseverance of the secondary structure elements in the protein complex, especially for the AfArsR arsenic binding site in the protein complex. Ultimately, the model generated by Robetta was chosen (Figure 5A) to be carried forward for further simulations as it is more structurally preserved compared to others. 

Having selected the Rosetta-generated model for further structure validation, we ran molecular dynamics (MD) simulations for 150 ns and compared four different structures of GEAR-G1 at different time scales of the MD simulation: 0 ns, 50 ns, 100 ns, and 150 ns (Figure 5B). It was observed that the C-terminal helix of GFP in GEAR-G1, which was visible at 0 ns, had completely transformed into a coil structure within 50 ns of simulation. While the structure of GFP in GEAR-G1 was well-maintained throughout the MD simulation, a conformational change in the region of residues 80, 81, and 82 can be observed. Additionally, some minor structural changes can also be seen in the AfArsR region. The Dictionary of Secondary Structure of Protein (DSSP) graph provided information regarding α-helix, β-sheet and loop content in the protein [37]. In the DSSP graph of GEAR G1 (Figure 5C), no major changes were observed except for a few minor changes in coils and short helix (Figure 5C, shown in red and cyan), possibly due to the formation of coils in the C-terminal of GFP in GEAR-G1. As shown in Figure 5D, the root means square deviation (RMSD) and radius of gyration (Rg) values are reasonably stable throughout the last 100 ns of MD simulation (i.e., 50–150 ns) with average values of 0.44 ± 0.04 nm and 2.36 ± 0.02 nm, respectively. The root mean square fluctuation (RMSF) plot (Figure 5D, bottom panel) indicated some noticeable fluctuations for residues 180–195, 215–225, and 245–255, which mostly represent the loop and coil regions of the AfArsR domain. The GFP domain is rather stable except for the highly flexible C-terminus. Overall, these computational structure analyses showed that the GEAR-G1 secondary structure elements were conserved with only small variations during the 150 ns simulation of GEAR-G1.

To investigate the correlated motions between residues of GEAR-G1 protein complex, we performed dynamic cross correlation matrix analysis on the trajectory obtained from the last 100 ns of MD simulation. The cyan and purple colors indicate positive and negative correlation motions between fluctuating residues, respectively. In Figure 5E, the diagonal, cyan-colored region shows a positive correlation of topologically proximate residues. Moreover, the analysis also suggests highly correlated intra-residual motion within GFP and AfArsR residues, as well as an inter-residual cross correlation between GFP and AfArsR residues.

**Figure 6:**
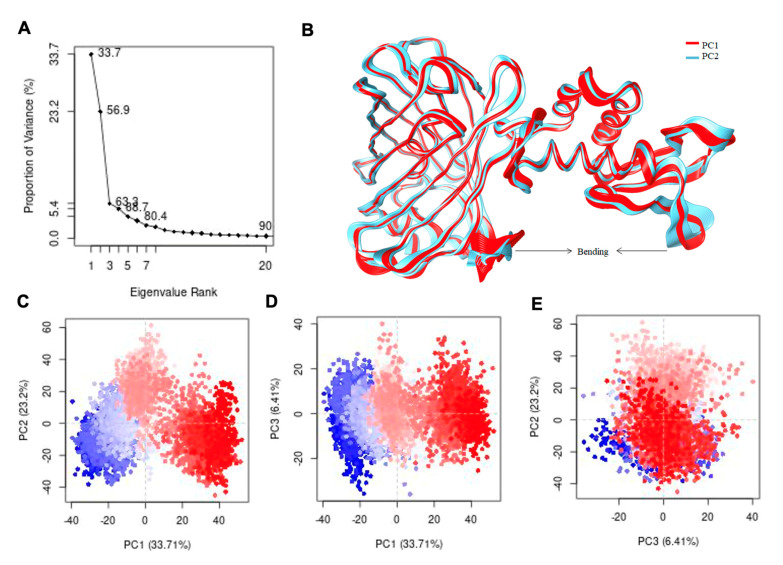
(**A**) Depiction of the proportion of variance (%) against its Eigenvalue rank. (**B**) Visualization of dynamics of PC1 for depiction of the fluctuating regions. (**C**–**E**) Projection of the trajectory formed by the first three principal component analyses based on k-means values.

To confirm the correlated dynamic nature of GEAR-G1, we performed principal component analysis (PCA) using the α-carbon (Cα) position covariance. The first PC (PC1) accounts for 33.7% variance, which shows the largest variation in protein dynamic. The second PC (PC2) captures 23.2% of variance in protein, which is the second most important direction, and it is orthogonal to the PC1 axis. Altogether, the first three PC components account for more than 63% of protein variance (Figure 6A). The first PC highlights a twisting motion of the whole AfArsR region with the GFP; the N-terminal region of GFP exhibits a pronounced twisting effect (Figure 6B). On the other hand, the PC2 displays a scissoring motion (bending motion) of the AfArsR and GFP regions, which depicts the negative correlation motion (Figure 6C–E). Based on PC1, PC2 and PC3 variances, the conformers are clustered into four groups (colored as blue, cyan, pink, and red) based on the k-means algorithm values (Figure 6C–E) [38]. The red color region shows that there is less movement, the white region shows intermediate movement, while the blue region has the most significant movement or flexibility. The PCA identified the dominant regions of protein during simulation. The GFP domain (shown in red color) is pretty much stable, with the only exception being the flexible loops at the C-terminus. On the contrary, the AfArsR domain showed sufficient fluctuations as represented in blue color in Figure 6B, suggesting that the AfArsR domain is structurally highly flexible.

## 4. Conclusions

Over the past two decades the development and application of genetically encoded biosensors has steadily broadened the scope of scientific questions that cell biologists can ask and answer [39,40]. With the rapid expansion of the biosensor toolkit, a wider range of molecules now can be detected using FP-based biosensors to benefit more areas of biological sciences [26,29,41,42,43,44]. Here, we have further broadened this target range of genetically encoded biosensors by establishing the ‘GEAR series’ of FRET-based and single FP-based genetically encoded As^3+^ biosensor prototypes. Using rational engineering approaches, we successfully improved the performance of these biosensors. We also used the newly developed FRET-based biosensor GEAR-CV1 to study the role of the AfArsR As^3+^ binding site residues using site-directed mutagenesis and fluorescence spectroscopy, providing further support for the conclusions of previous studies [15,16]. We thus expect this could serve as a generally useful approach for investigation of mutational effect on target binding as long as the binding-induced conformational change can be measured by an observable FRET change. Using computational structure prediction and simulations, we further obtained valuable structural and mechanistic insights for sensor optimization. Future effort should be directed towards experimental structure determination in order to reveal and provide insight into the detailed sensing mechanism.

The newly developed GEAR biosensor prototypes, together with the EcArsR-based SenALiB [14], are promising templates for future development of high-performance As^3+^ biosensors. Further optimization using directed evolution is likely to yield new variants with substantially improved sensitivity for in vitro and in vivo detection of As^3+^. Engineered and improved biosensors with proper brightness, sensitivity, and selectivity are expected to unlock new possibilities for investigation of cellular signaling associated with As^3+^ dynamics and toxicity in the native cellular environment. Moreover, these biosensors serve as the first examples using the ArsR transcriptional regulator for genetically encoded biosensor engineering. The transcriptional factor ArsR superfamily has a diversified range of binding targets [45]; therefore, it represents a promising resource to be exploited for the future engineering of fluorescent biosensors.

## Data Availability

The data supporting this research are available upon request.
